# Early Screening and Risk Factors of Autism Spectrum Disorder in a Large Cohort of Chinese Patients With Prader-Willi Syndrome

**DOI:** 10.3389/fpsyt.2020.594934

**Published:** 2020-11-26

**Authors:** Xuejun Kong, Junli Zhu, Ruiyi Tian, Siyu Liu, Hannah T. Sherman, Xiaoying Zhang, Xiaojing Lin, Yan Han, Zhi Xiang, Madelyn Koh, Clara Hobbie, Bryan Wang, Kevin Liu, Jun Liu, Yueping Yin, Guobin Wan

**Affiliations:** ^1^Athinoula A. Martinos Center for Biomedical Imaging, Massachusetts General Hospital, Charlestown, MA, United States; ^2^Fisher College, Boston, MA, United States; ^3^Hangzhou Seventh People's Hospital, Hangzhou, China; ^4^Prader-Willi Syndrome Care and Support Center, Hangzhou, China; ^5^Institute of Dermatology and Hospital for Skin Disease, Chinese Academy of Medical Science and Peking Union Medical College, Nanjing, China; ^6^Carleton College, Northfield, MI, United States; ^7^Brandeis University, Waltham, MA, United States; ^8^Shenzhen Maternity & Child Healthcare Hospital, Shenzhen, China

**Keywords:** Prader-Willi Syndrome (PWS), Autism Spectrum Disorder (ASD), autism like phenotype (ALP), weight, height

## Abstract

Previous studies regarding the prevalence of Autism Spectrum Disorder (ASD) in patients with Prader-Willi Syndrome (PWS) have implicated heterogenous findings. Additionally, the early screening of ASD high-risk population for ASD and identifying ASD risk factors in PWS patients have not been explored. This study included 218 Chinese PWS patients aged 3 months to 18 years old. 78% of subjects were identified as high risk for ASD by ASQ-3 Communication domain score for those younger than 3 years of age and 84% of subjects were classified as high risk for ASD by the GARS-3 for those aged 3 years and older. Among PWS clinical measurements, under-height (*P* = 0.0186), overweight (*P* = 0.0248), and obstructive sleep apnea (*P* = 0.0259) were each significantly correlated with ASD risk. These risk factors and their internal relationship with ASD or ASD traits warrant further studies.

## Introduction

Autism Spectrum Disorder (ASD) is a neurodevelopmental disorder characterized by impairment in social communication and repetitive behaviors and restricted interests (1). Among patients with diagnosed ASD, 80–90% of cases are idiopathic, while 5–20% of cases are due to known genetic disorders, so-called syndromic ASD (2–5).

Prader-Willi Syndrome (PWS) is a type of syndromic ASD and rare genetic disorder that can be caused by one of the three genetic abnormalities: a paternal deletion of the 15q11.2–q13 region, maternal uniparental disomy (mUPD), or an imprinting defect (ID) (6, 7). Studies suggest that the prevalence of ASD in PWS ranges from 12.7 to 40% with significant overlaps in their clinical features (8–14). The early identification and screening of individuals with PWS that are at risk for ASD will help facilitate early intervention, which can significantly impact their prognosis, thus highlighting the need for further studies. In addition, the sample sizes in these studies were limited and the milder and broader phenotypes were not explored nor included in the reported prevalence.

Early screening of those at risk for ASD is crucial to facilitating the diagnosis of ASD in PWS and for identifying those outside the categorical boundary of a diagnosis, so-called broad autism phenotypes (BAP) or autism-like phenotypes (ALP). To differentiate from the term “BAP” in reference to family trees, we hereby refer to such subjects as those with ALP, defined analogously as individuals displaying features of autism but does not meet the diagnostic criteria of ASD currently or later in development. The early identification of the at-risk population and associated risk factors will allow better prevention and treatment strategies before or after a formal diagnosis was made and allow such individuals to understand their social or behavioral deficits that require treatment, which would often otherwise remain untreated. There has been an increasing number of studies involving individuals displaying BAP in recent years due to its significance in identifying genetic clusters that may cause ASD symptoms. While BAP has been found in individuals without known relatives with ASD, past studies involving BAP have been primarily focused on family members of idiopathic ASD individuals (15–20). Notably, no existing study has examined BAP or ALP in syndromic ASDs, such as PWS. In contrast to idiopathic ASD, abnormalities of many syndromic ASDs are mostly caused by *de novo* mutations. For example, PWS is almost exclusively caused by *de novo* mutations, such that there are neither genetic predispositions nor an increased risk for developing PWS among family members. Nonetheless, individuals with these genetic syndromes have a significant risk of either fully developing ASD (ranging from 11 to 61%) or manifesting milder ASD phenotypes which have not yet clearly reported (11, 21–24). Examining genetic and non-genetic risk factors of ASD and ALP in syndromic ASDs additionally provides insights through a novel pathway to elucidate ASD pathogenesis and increase understanding of the shared pathophysiological mechanisms underlying ASD and its related phenotypes.

We included 218 PWS individuals in this study to screen for those at risk for ASD and study the associated risk factors. We aim to facilitate the early diagnosis and recognition of ASD and its milder phenotypes, allowing early prevention, and intervention implantations in hopes of promoting a better prognosis. This study may also pave way for future studies to explore ASD genetic and epigenetic mechanisms among individuals with PWS and other syndromic ASDs.

## Methods

### Participants and Procedures

Study participants were recruited through the PWS Care & Support Center located in Zhejiang, China, which is a PWS association with a nationwide registry and services. Participants are included based on having a genetically confirmed PWS diagnosis and do not have any other known severe genetic disorders, including Fragile X syndrome, Angelman syndrome, tuberous sclerosis, and Down syndrome. Participants are excluded if they have not been genetically confirmed with Prader-Willi syndrome or if they have other known severe genetic disorders as mentioned above. No restrictions on age were imposed for subject recruitment.

Ethical approval was issued by the Internal Review Board (IRB) of the Dermatology Institute of Chinese Academy of Medical Science in Nanjing. According to the IRB requirements, written informed consent was obtained from competent adult subjects or the parents or legal guardians of children and adults with cognitive impairment.

After enrollment in the study, the parents of the subjects completed one of two surveys provided by the research team. Subjects 3–36 months in age were given the Ages and Stages Questionnaire-3 (ASQ-3), while subjects 36 months−18 years were given the Gilliam Autism Rating Scale-3 (GARS-3). The surveys were submitted to the PWS Care & Support Center, where the results were de-identified before being sent for data analysis. Surveys were provided to 465 subjects; 328 subjects returned the survey, but 218 were valid with a fully complete survey. One hundred ten surveys with missing information were excluded for data analysis.

### Measures

Subject demographics were collected, including age, sex, genotypes, height, weight, and BMI. Clinical variables were collected for each subject. These included obstructive sleep apnea (OSA), hypotonia, hypoxia, epilepsy, scoliosis, hip joint dislocation, undescended testicle, growth hormone usage, nasal tube feeding, feeding difficulty, reduced sensitivity to pain, light-colored hair, unusually fair skin, narrow forehead, and weak crying.

In order to classify study subjects of either low or high risk for developing ASD, families were provided with one of two published and widely respected assessments, depending on their ages:

#### Ages and Stages Questionnaires, 3rd Edition (ASQ-3)

The ASQ-3 was handed or mailed to the parents of the 103 participants younger than 36 months old. It is one of the most widely available development, communication, and behavior screening tools for young children (25, 26). The ASQ-3 provides parents with information about the developmental status of their young child across five developmental domains: communication, gross motor, fine motor, problem-solving, and personal-social. Hardy et al. screened 2,848 toddlers with the ASQ-3 and M-CHAT-R across 20 pediatric sites, used the “monitor and/or fail” cutoff on any domain, the ASQ-3 identified 87% of the children who screened positive on the M-CHAT-R with follow-up and 95% of those diagnosed with an ASD (27). Beacham et al. tested 124 diagnosed with ASD, ASQ-3 identified 82% by the Communication domain and 85% identified by M-CHAT-R, which further confirmed the compatibility of ASQ-3 and M-CHAT-R (28). In this study, the patients who scored “pass” in the Communication domain of ASQ-3 were classified in the low-risk group of ASD, while those who scored “monitor” or “fail” were put in the high-risk group of ASD.

#### Gilliam Autism Rating Scale, Third Edition (GARS-3)

Patients aged 36 months or older, received the GARS-3 from the PWS Care & Support Center. GARS-3 is a norm-referenced screening instrument used to identify persons with autism spectrum disorders for age 3–22, third edition since 1995 (29). It has proven to have a high rate of validity and reliability, which makes it highly utilized in the psychology field (30, 31). It consists of 56 items describing characteristic behaviors of individuals with autism. The items are grouped into six subscales: restrictive, repetitive behaviors (RB), social interaction (SI), social communication (SC), emotional responses (ER), cognitive style (CS), and maladaptive speech (MS).

The results of GARS-3 came in the form of the Autism Index scores, which were obtained by converting the sums of the subscale scores into index scores. The higher the Autism Index score, the greater the probability that an individual had ASD. GARS-3 provides four levels of probability of having ASD: level 0 with an Autism Index ≤ 54, “unlikely;” level 1 with an Autism Index between 55 and 70, requiring “minimal support;” level 2 with an Autism Index between 71 and 100, “very likely” and requiring substantial support; level 3 with an Autism Index ≥ 101, “very likely,” and requiring very substantial support (29). In this study, the patients with ASD probability at level 0 and level 1 were classified in the low-risk group of ASD, while those whose probability of having ASD at level 2 and level 3 were in the high-risk group of ASD.

### Data Analysis

We first calculated the mean and standard deviation for each continuous variable and examined their distributions, including the ASQ-3 and GARS-3 scores. We also examined the frequency of each categorical variable included. Then, we identified the correlation between a subject's classification on the ASQ-3 Communication domain and the other four ASQ-3 domains. Moreover, we calculated the prevalence of PWS patients who scored “Fail” or “Monitor” in the Communication domain as well as their prevalence of scoring “Fail” and “Monitor” in the other four 4 domains and tested whether this significantly differed using Pearson's Chi-squared test. Lastly, we used Pearson's Chi-squared test to examine the correlation of clinical variables with the ASD risk level. A confidence level of 95% was used to determine statistical significance for all tests.

## Results

### Demographic Features of PWS Patients

This study included 218 patients with genetically confirmed Prader-Willi syndrome aged 48.4 ± 42.3 months and consisting of 97 (45.5%) females and 121 (55.5%) males. A detailed summary of overall study population characteristics is provided in Table 1, including age, sex, height, body mass index (BMI), genotypes, and observed prevalence of obstructive sleep apnea (OSA).

**Table 1:** Summary of subject demographics and basic characteristics.

	**Feature**	***n* (%)**
Age	<3 yrs.	113 (51.8)
	3 yrs.	105 (48.2)
Sex	Female	97 (44.5)
	Male	121 (55.5)
Genotype	Paternal deletion	180 (82.6)
	mUPD	21 (9.6)
	Others	17 (7.8)
Height	Under-height	132 (60.5)
	Normal	70 (32.1)
	Over-height	16 (7.3)
BMI	Underweight	59 (27.1)
	Normal	58 (26.6)
	Overweight	101 (46.3)
OSA		46 (21.1)

**Figure 1:**
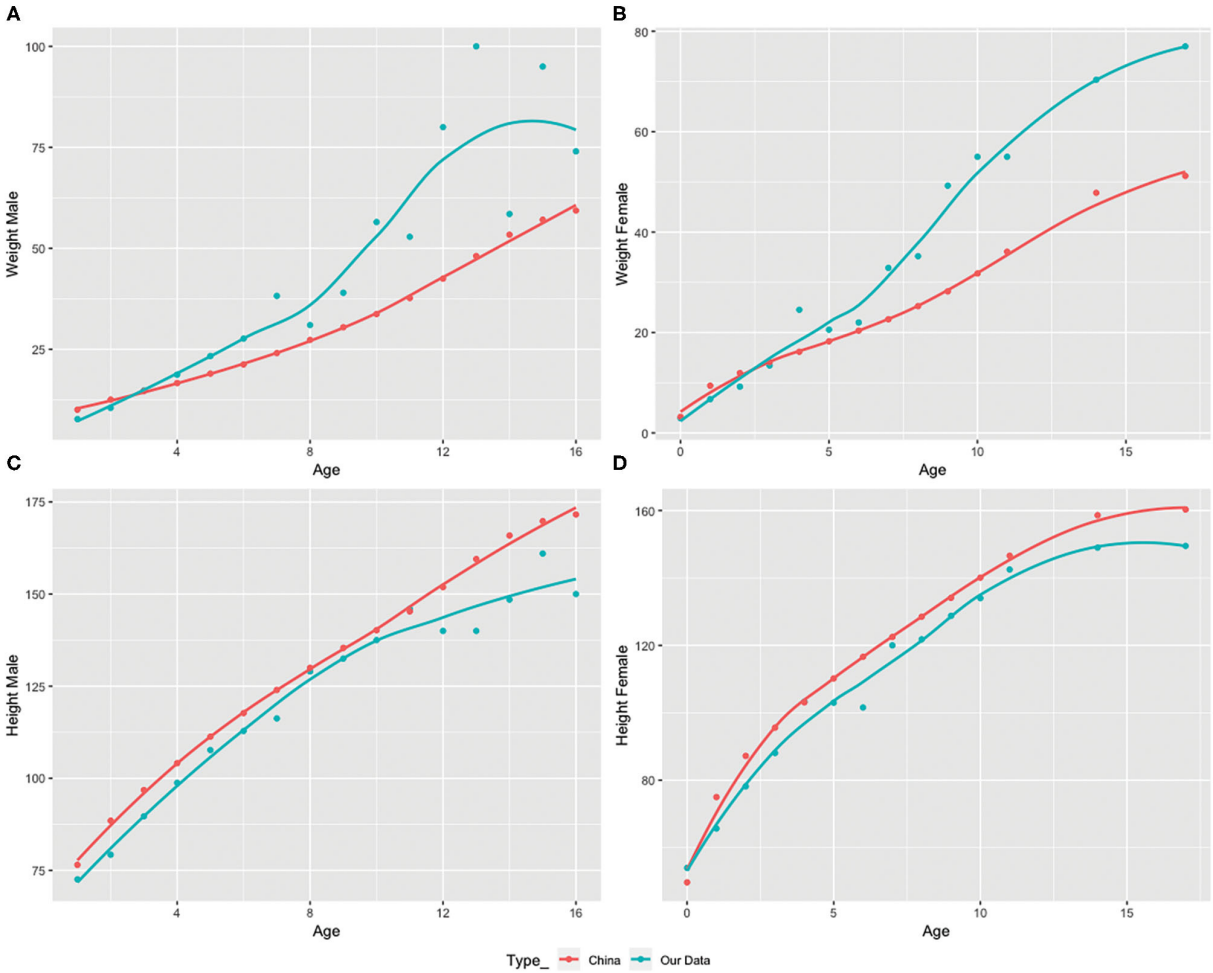
Weight and height variations with age in the study subject population compared to the 50th percentile value according to China's child growth weight standard from age 1 to 16 in males and females provided by the World Health Organization (WHO). **(A,B)** Comparison of observed and standard 50th percentile weight in males and females by age. **(C,D)** Comparison of observed and standard 50th percentile height in males and females by age.

Figure 1 demonstrated the changes in weight and height with age according to standards published by the World Health Organization (32, 33). Among patients under 3 years, 29.20% have a BMI lower than the 3rd percentile of their age group and 50.44% have a BMI lower than the 15th percentile (underweight); however, among patients 3 years of age or older, 72.38% have a BMI higher than the 97th percentile and 83.81% have BMI higher than 85th percentile (overweight). Since all patients were Chinese, weight, and height demographics were further compared to the child growth standard in China (34). Among male subjects, the observed weight exceeded the 50th percentile at 3 years and older while female subjects' weight exceeded the 50^th^ percentile at 4 years and older (Figures 1A,B). Height stayed under the 50^th^ percentile with the advancement of age in both genders (Figures 1C,D).

### ASQ-3 Total and Sub-scores and ASD Risks

Table 2 showed the domain-specific results of ASQ-3. Using communication domain as an indicator, high-risk group was identified as 77.9% included “fail” (58.4%) and “monitor” (19.5%), while low-risk group was 22.1% who had “pass.” Other domains fell into similar predominant “fail” and “monitor” proportion as the communication domain.

**Table 2:** Overview of ASQ-3 subscale scores among all subjects by five domains.

**Domains**	**Mean ± SD**	**Fail (*n* %)**	**Monitor (*n* %)**	**Pass (*n* %)**
Communication	22.3 ± 16.2	66 (58.4)	22 (19.5)	25 (22.1)
Gross motor	13.6 ± 17.7	100 (88.5)	1 (0.88)	12 (10.6)
Fine motor	21.7 ± 16.3	87 (77)	13 (11.5)	13 (11.5)
Problem solving	19.3 ± 16.3	71 (62.8)	22 (19.5)	20 (17.7)
Personal social	22.5 ± 15.7	81 (71.7)	19 (16.8)	13 (11.5)

### GARS-3 Total and Sub-scores and ASD Risks

Based on the GARS-3, the high-risk group was identified as 83.8%, including level 2 (68.6%) and level 3 (15.2%), while the low-risk group was 16.2%, including levels 0 and 1; and a detailed summary of the distribution of each functional level among subjects is shown in Table 3. Table 4 lists the summary of results based on GARS-3 subscales.

**Table 3:** Summary of GARS-3-based risk and functional levels of autism among *n* = 111 (50.9%) subjects.

**Risk category**	**Functional level**	**Distribution (*n* %)**
Low risk	level 0	1 (1.0)
	level 1	17 (15.2)
High risk	level 2	76 (68.6)
	level 3	17 (15.2)

**Table 4:** Overview of GARS-3 subscale scores among all subjects.

**Feature (*n* = 149, 68.3%)**	**Mean ± SD**
Restricted/repetitive behaviors (RB)	17.4 ± 8.91
Social interaction (SI)	11.5 ± 9.23
Social communication (SC)	14.0 ± 6.7
Emotional responses (ER)	13.6 ± 4.74
Cognitive style (CS)	10.5 ± 4.12
Maladaptive speech (MS)	7.32 ± 5.07
Total score (4 subscales)	31.1 ± 8.68
Total score (6 subscales)	50.5 ± 11.9

### Correlation of PWS Clinical Indices and ASD Risk

Further Chi-squared tests were performed to examine the statistical significance of the frequency differences of clinical indices variables between the high risk and low-risk groups of ASD. As shown in Table 5, statistical significance (*P* < 0.05, 95% CI) was found in under-height (*P* = 0.0186), overweight (*P* = 0.0248), obstructive sleep apnea (*P* = 0.0259) of patients over 3 years old.

**Table 5:** Univariate analysis of clinical indices risk factors across age groups.

	**Characteristics**	**ASQ-3 (<3 years old)**		**GARS-3 (>3 years old)**	
	**OR (95% CI)**	*P-*value^*^	**OR (95% CI)**	*P-*value^*^	
Sex	Male	Reference	0.2159	Reference	0.3129
	Female	0.57 (0.23, 1.40)		1.72 (0.59, 4.97)	
BMI	Normal	Reference	–	Reference	–
	Underweight	1.08 (0.41, 2.82)	0.9071	3.27 (0.58, 33.84)	0.2187
	Overweight	1.87 (0.58, 7.26)	0.4520	3.60 (1.57, 7.16)	0.0248^*^
Height	Normal	Reference	–	Reference	–
	<15th percentile	0.80 (0.33, 1.70)	0.6908	4.04 (1.48, 11.35)	0.0186^*^
	>85th percentile	2.00 (0.29, 20.81)	0.3255	0.97 (0.39, 2.95)	0.9683
Genotype	Paternal deletion	Reference	0.1670	Reference	0.8338
	Non-paternal deletion	2.44 (0.67, 8.96)		1.18 (0.24, 5.81)	
Growth hormone usage	No	Reference	0.8938	Reference	0.0705
	Yes	1.07 (0.38, 3.05)		0.35 (0.11, 1.13)	
Sleep apnea	No	Reference	0.1818	Reference	0.0259^*^
	Yes	2.75 (0.59, 12.83)		7.70 (0.98,60.68)	
Epilepsy	No	Reference	0.6687	Reference	0.7310
	Yes	0.59 (0.05, 6.80)		0.75 (0.14, 3.88)	
Amniotic fluid level	Low	Reference	–	Reference	–
	Normal	2.28 (0.67, 7.69)	0.3603	0.60 (0.14, 2.47)	0.7545
	High	1.35 (0.40, 4.57)	0.3603	0.79 (0.15, 4.00)	0.7545
Delivery method	Spontaneous	Reference	0.7719	Reference	0.5714
	C-section	0.85 (0.28, 2.55)		1.49 (0.37, 5.99)	
**Neonatal or infancy characteristics**					
Nasal tube feeding	No	Reference	0.1673	Reference	0.0654
	Yes	1.93 (0.75, 4.96)		5.75 (0.72, 45.78)	
Narrow forehead	No	Reference	0.2470	Reference	0.6476
	Yes	1.70 (0.69, 4.17)		1.28 (0.44, 3.74)	
Light-colored hair	No	Reference	0.7198	Reference	0.6884
	Yes	1.18 (0.47, 2.93)		0.81 (0.29, 2.24)	
Hypoxia	No	Reference	0.4446	Reference	0.8241
	Yes	1.44 (0.56, 3.72)		0.89 (0.32, 2.46)	

* *P-values are for any difference between high risk and low risk using t-test or χ^2^-squared test as appropriate*.

## Discussion

Among the 218 Chinese PWS participants enrolled in this study, we found that 78% of subjects younger than 3 years old were identified as high risk for ASD via ASQ-3, while 84% of subjects aged 3 years or older were identified as high risk for ASD via GARS-3. There have been only limited studies that reported the prevalence of ASD in PWS patients. Veltman et al. conducted a systematic review in 2005 and proposed that the prevalence of ASD in PWS patients is 25.3% (35). Dykens et al. reported in 2017 that 12.3% of 146 participants (aged 4–21 years old) with PWS were identified as having ASD, using the Autism Diagnostic Observation Schedule, Second Edition (ADOS-2). The large variations reported in studies on the prevalence of ASD in PWS patients are likely due to limited sample size as a result of the rarity of the disorder, variations in age and ethnicity, and different sensitivity and specificity of the methods used for evaluation. Our study participants were exclusively Chinese; 50% of them were <3 years old. Both ASQ-3 and GARS-3 were used as screening tools instead of diagnostic tools. These two measurements were selected not only because they are age/psychometrically appropriate for our two age groups, but also because they best serve our aims of early screening in this survey study. For instance, the ASQ-3 has comparable sensitivity and specificity with the well-known M-CHAT (27); in addition, the ASQ-3 covers a broader age range and includes five developmental domains. Meanwhile, the GARS-3 consists of 56 items describing characteristic behaviors of individuals with autism; its six subscales include emotion responses and cognition style in addition to the two other ASD core symptoms resulting in a broader coverage in comparison to the ADOS. Additionally, there has been a growing recognition of the potential relationship between Prader-Willi Syndrome and Autism Spectrum Disorder (10). Through clinical observation, it is noticed that some of the behavioral features of Prader-Willi Syndrome overlap with those found in individuals with Autism Spectrum Disorder (8). Thus, we believe that our results from these screening methods are generalizable to the early screening of those with ALP in PWS patients while also providing a better reflection of cross-sectional functional levels and other associated deficits which may pose significant impact to their education, career, and quality of life. Ideally, a parallel and longitudinal study using standard diagnostic tools such as ADOS-2 should be done to confirm the assumption. Nonetheless, we hope that our study provides insight into the current tools used to identify at-risk populations at an early stage via easily applied screening methods in hopes that they will provide for more appropriate, comprehensive diagnostic testing. These preliminary results shed light for future research on autism genotype-phenotype correlation and ASD pathogenesis among patients with syndromic ASDs which will, in-return, help the broader at-risk population identify their needs and receive targeted interventions at an earlier stage. For instance, psychosocial and education programs can be tailored and individualized based on the cognitive style, emotion response and weakness areas, and implemented earlier to achieve future success in education, employment and independent life.

It is essential to recognize the early signs of ASD amongst younger PWS patients. Research suggests that PWS patients with ASD generally show deviant language production, obsessive interests, dependency on patterns, routine and/or rituals, self-mutilation, and unpredictable behavior (10). However, these autism phenotypes are less prominent and thus more difficult to detect at an early age. Therefore, it is essential to develop effective early screening tools and assess risk factors. ASQ-3 is a broad development screener suitable for newborn to preschoolers. From Hardy, Beecham, and others' studies, the ASQ-3 communication domain has been identified as a sensitive screener for ASD and is compatible with M-CHAT-R with up to 90% sensitivity in 16–36 months children. Beecham et al. reported that ASQ-3 combined with Mullen Scales of Early Learning (MSEL) improved the sensitivity and specificity of screening, which could be the direction for our future studies. The use of ASQ-3 in combination with MSEL will be particularly helpful for recognizing early signs of ASD in those younger than 16 months old as the current screening and diagnostic tools, M-CHAT-R and ADOS-2, does not include this age range and does not have validity at this age, respectively. Thus, ASQ-3 is a promising early screening tool for ASD and ALP and warrants further studies. Although the specificity (of what? ASQ-3?) might not be as high as its sensitivity, it is imperative to identify the broader high-risk populations early on and get an early start on corresponding intervention and prevention strategies to improve the prognosis of later ASD diagnosis or ALP in PWS patients. While a formal diagnosis is always essential, it might not always be readily available and could be frequently delayed particularly in underserved areas and countries.

Lastly, our study examined the risk factors contributing to the autism phenotypes in patients with PWS with predictive values. We analyzed multiple clinical indices to identify their correlations with ASD risk. We found that under-height, overweight, and having OSA, may predict ASD risk; each factor has a different extent of correlation, but all have not yet been reported by previous literature. Among these three factors, under-height demonstrated the most significant correlation with higher ASD risk (*P* = 0.0186), which we believe is an important finding. Unlike overweight, under-height is a consistent feature of PWS subjects independent of age (Figure 1B), while overweight only becomes prominent after 3 years of age (Figure 1A). Under-height could potentially serve as a better predictive indicator of ALP and ASD development in comparison to overweight. Interventions that lead to increased height in PWS may substantially reduce ALP expression later in life and improve long-term prognosis. Growth hormone (GH) replacement was found not only to increase height, but also to decrease body fat and improve cognition, motor, and mental function, which is currently considered the most effective treatment for PWS (36–39).

This study showed that growth hormone treatment correlated with a lower risk for ASD, although it failed to demonstrate statistical significance (*P* < 0.1), possibly due to smaller powers. We found that overweight is also associated with being at high risk of ASD (P < 0.05); thus, early weight management with strict diet control on top of GH treatment might greatly help prevent ALP and ASD expression. OSA is a prevalent complication of PWS as the Sedky et al. found that the prevalence of OSA among 224 PWS children was 79.91% Sedky et al. (40). OSA is also significantly correlated with ASD risk in this study (*P* < 0.05). The prevalence of this comorbidity among PWS patients is higher than in the general population (40–42). We suggest that early diagnosis and treatment of sleep disorders among PWS patients could reduce their ALP and ASD expression and improve their overall prognosis.

There are several limitations of this study worth considering. (1) Although we used norm-referenced screening instruments ASQ-3 and GARS-3 to study ASD risk, we didn't use more specific diagnostic tools such as ADOS or ADI-R or conduct a longitudinal study to further differentiate those with ASD or ALP; however, we intend to include these diagnostic tools in our future studies; (2) the wide age range of participants in this study and lack of control in recruitment for IQ, psychiatric and medical conditions, and comorbidities resulted in high subject population heterogeneity, which could potentially interfere with the results; (3) parents or caregivers who filled out the questionnaire could potentially have a subjective bias, which might interfere with the result. Despite these limitations, the findings from this study are still meaningfully significant to direct further projects.

## Conclusions

The correlation between PWS and ASD was found to be much higher than previously reported because ALP, the milder forms of autism phenotypes, were included–an aspect that has not yet been explored and warrant further study. The use of highly sensitive and easily applied screening tools for early identification of high-risk populations and related risk factors for ASD in PWS patients are of special value for facilitating early diagnosis and early intervention leading to a better overall prognosis. This applies not only to those meeting ASD criteria later on but also for those broader populations with ALP. Through the identification of ASD core symptoms and related areas, more individualized intervention programs can be designed to help individuals with ASD achieve future educational and professional success. The results of this study provide a proof-of-concept that can serve as an empirical basis for further genetic research on the mechanism of ASD and ALP development.

## Data Availability Statement

The datasets presented in this study can be found in online repositories. The names of the repository/repositories and accession number(s) can be found at: https://figshare.com/, doi: 10.6084/m9.figshare.12800792.

## Ethics Statement

The studies involving human participants were reviewed and approved by Dermatology Institute of Chinese Academy of Medical Science. Written informed consent to participate in this study was provided by the participants' legal guardian/next of kin.

## Author Contributions

XK conceived the concept, designed and led the study and data analysis, drafted the first version and revised the manuscript. JZ and RT contributed to data analysis, tables and figures and writing. SL, HS, CH, and BW contributed to revision of the manuscript. XZ and XL assisted in obtaining consent from study participants, subject coordination, survey monitoring and data collection. YH, ZX, and YY contributed to the IRB application and approval. MK and JL contributed to the coordination of IRB application. GW contributed to survey protocol and manuscript revision. All authors have approved the submission.

## Conflict of Interest

The authors declare that the research was conducted in the absence of any commercial or financial relationships that could be construed as a potential conflict of interest.
